# Role of Young Child Formulae and Supplements to Ensure Nutritional Adequacy in U.K. Young Children

**DOI:** 10.3390/nu8090539

**Published:** 2016-09-02

**Authors:** Florent Vieux, Chloé M. C. Brouzes, Matthieu Maillot, André Briend, Régis Hankard, Anne Lluch, Nicole Darmon

**Affiliations:** 1 MS-Nutrition, Marseille 13005, France; matthieu.maillot@ms-nutrition.com; 2 Danone Nutricia Research, Centre Daniel Carasso, RD128, Palaiseau 91767, France; chloe.brouzes@danone.com (C.M.C.B.); anne.lluch@danone.com (A.L.); 3 Center for Child Health Research, University of Tampere School of Medicine and Tampere University Hospital, University of Tampere, Tampere 33101, Finland; andre.briend@gmail.com; 4 Department of Nutrition, Exercise and Sports, Faculty of Science, University of Copenhagen, Rolighedsvej 30, Frederiksberg DK-1958, Denmark; 5 INSERM-U 1069, Univ. F. Rabelais, Tours F-37000, France; regis.hankard@inserm.fr; 6 Aix Marseille Univ, INSERM, INRA, NORT, Marseille 13005, France; nicole.darmon@univ-amu.fr

**Keywords:** young child formula, supplements, individual diet modeling, diet, EFSA, U.K.

## Abstract

The European Food Safety Authority (EFSA) states that young child formulae (YCFs) “cannot be considered as a necessity to satisfy the nutritional requirements” of children aged 12–36 months. This study quantifies the dietary changes needed to ensure nutritional adequacy in U.K. young children who consume YCFs and/or supplements and in those who do not. Dietary data from 1147 young children (aged 12–18 months) were used to identify, using linear programming models, the minimum changes needed to ensure nutritional adequacy: (i) by changing the quantities of foods initially consumed by each child (repertoire-foods); and (ii) by introducing new foods (non-repertoire-foods). Most of the children consumed neither YCFs, nor supplements (61.6%). Nutritional adequacy with repertoire-foods alone was ensured for only one child in this group, against 74.4% of the children consuming YCFs and supplement. When access to all foods was allowed, smaller food changes were required when YCFs and supplements were initially consumed than when they were not. In the total sample, the main dietary shifts needed to ensure nutritional adequacy were an increase in YCF and a decrease in cow’s milk (+226 g/day and −181 g/day, respectively). Increasing YCF and supplement consumption was the shortest way to cover the EFSA nutrient requirements of U.K. children.

## 1. Introduction

Between six and 24 months of age, the transition from an exclusively milk-based diet to a more diverse and adult-like diet has to cover growing nutrient and energy needs [1], while allowing the introduction of new textures and tastes [2,3]. During this period, diets may include different milks (e.g., breast milk, cow’s milks) or commercial formulae. Young child formulae (YCFs) are defined by the European Food Safety Authority Panel on Dietetic Products, Nutrition and Allergies (EFSA NDA Panel) as formulae intended for young children (12–36 months), including formulae based on protein sources other than cow’s milk [4]. Fortified with nutrients, such as essential fatty acids, iron and vitamin D, YCFs are designed to support the nutritional needs of young children as part of a balanced diet [5,6].

In 2013, the EFSA NDA Panel derived levels of nutrients considered adequate for most infants and young children by reviewing reference values set by the Scientific Committee for Foods in 1993 [7] in light of more recent recommendations [8]. The same report indicated that YCFs were “one of several means to increase *n*-3 PUFA, iron, vitamin D and iodine intakes in infants and young children living in Europe with inadequate or at risk of inadequate status of these nutrients”. The report also states that there is “no unique role of young-child formulae in satisfying the nutritional requirements of young children” [8]. The potential contribution of YCFs to the diets of young children in Europe thus needs to be clarified. There are several ways in which nutrient gaps in the child’s food diversification phase can be filled. These include changing dietary habits, consuming fortified foods and drinks (such as YCFs) and/or taking supplements. The choice will depend on local habits, individual acceptability, the cost and accessibility of products and the quality and quantity of the foods and drinks already being consumed.

Linear programming can be used to support appropriate complementary feeding advice based on locally-available foods [3,9,10,11]. Taking into account individual food patterns and preferences, linear programming can translate specific nutrient recommendations into realistic individual food choices [12]. In the present study, individual diet modeling was used to determine the dietary changes needed to attain nutritional adequacy in U.K. young children who consume YCFs and/or supplements and in those who do not. A nutritionally-adequate diet is defined as a diet covering a set of nutrient recommendations, here the EFSA nutrient requirements for 12–18 months. The hypothesis was that the consumption of YCFs and/or supplements was not strictly necessary to ensure nutritional adequacy, although it might facilitate it.

## 2. Materials and Methods

### 2.1. Dietary Survey and Study Sample

Dietary data were taken from the Diet and Nutrition Survey of Infants and Young Children (DNSIYC). This descriptive, cross-sectional, national survey conducted in 2011 provides detailed information on food and drink consumption on 4 consecutive days in a representative sample of 2683 infants and young children aged 4–18 months living in the U.K. The design, methodology and results of DNSIYC have been described elsewhere [13]. Full ethical approval for the initial survey was received from the Cambridgeshire 4 Research Ethics Committee on 18 January 2010.

To target the age group for which YCFs are designed, only children older than 12 months (*n* = 1275) were selected for this study. Data from children with less than 4 food-diary days completed (*n* = 35), or with an energy intake above or below 3 standard deviations (SD) from the mean (gender separated; *n* = 9) [14], or who consumed specialized formulae for allergy or lactose intolerance (*n* = 61) were ignored. Given that changes in YCF powder consumption cannot be considered alone (as it has to be associated with water), records only mentioning powder YCFs (*n* = 23) were also excluded, yielding a final sample size of 1147 children.

The sample was divided into four groups depending on the consumption of YCFs and/or supplement use: “no YCF, no Suppl”, “no YCF, Suppl”, “YCF, no Suppl” and “YCF & Suppl”.

### 2.2. Food Database

Energy and nutrient contents of foods were taken from the Department of Health’s Nutrient Database [15] and from information provided by manufacturers. Following the classification of foods provided by the DNSIYC study report, individual food items consumed (*n* = 2215 food items) were divided into 11 food categories and 26 food subcategories (Table S1). Supplements had their own category and were mostly multi-vitamins, with 61% of them including vitamins A, C and D. YCFs were included in the dairy products category. Compared with the average consumed cow’s milk, the average consumed YCFs contained lower levels of proteins, iodine, vitamin B12, riboflavin and SFA and higher levels of vitamin E, vitamin C, *n*-3 and *n*-6 fatty acids, iron and vitamin D.

### 2.3. Diet Quality Indicators

The nutritional quality of each diet was assessed with the mean adequacy ratio [16,17,18], using 22 beneficial nutrients in the calculation and estimated as follows (1):

(1) $$\mathrm{Mean}\;\mathrm{Adequacy}\;\mathrm{Ratio}=\frac{1}{22}\times \overset{22}{\underset{\mathrm{bn}=1}{\sum }}\frac{{\mathrm{intake}}_{\mathrm{bn}}}{{\mathrm{RV}}_{\mathrm{bn}}}\times 100$$

where intake_bn_ is the daily intake of each beneficial nutrient bn and RV_bn_ is the reference value (RV) for that nutrient. As previously proposed [19,20], each ratio (100 × intake_bn_/RV_bn_) was truncated at 100, so that a high intake of one nutrient could not compensate for a low intake of another.

The potential renal solute load (PRSL) refers to the solutes of dietary origin that must be excreted by the kidneys when not used for growing new tissues or lost through non-renal routes [21,22,23]. In this study, PRSL was estimated as described by Bonnet et al. [24], as follows (2):

(2) $$\mathrm{PRSL}\;\left(\mathrm{mmol}\right)=5.7\times \mathrm{protein}\;\left(g\right)+\frac{\mathrm{sodium}\;\left(\mathrm{mg}\right)}{23}+\frac{\;\mathrm{potassium}\;\left(\mathrm{mg}\right)}{39}+0.55\times \frac{\mathrm{phosphorus}\;\left(\mathrm{mg}\right)}{31}$$

A diet too high in proteins, sodium, chloride, potassium and phosphorus, with limited water intake, leads to a high PRSL.

### 2.4. Individual Diet Modeling with Linear Programming

The principle of diet modeling and the parameters used for this study are described in detail in Appendix A. Briefly, the present modeling approach was used to design diets meeting a set of nutritional recommendations, while keeping as close as possible to the observed diet. Two linear programming models were run for each child. Both were intended to make nutritionally-adequate diets, but differed in the list of foods allowed. “Repertoire-only models” allowed the inclusion of only repertoire-foods, i.e., foods recorded in an individual child’s four-day food diary. “All-foods models” allowed both repertoire-foods and non-repertoire-foods, i.e., all foods recorded in at least one food diary of the survey. “Repertoire-only models” were used to assess the feasibility of designing nutritionally-adequate diets with repertoire-foods only [25], while “all-foods models” were used to identify the food changes needed to attain nutritional adequacy with minimum changes to the existing diet [12]. Nutritional adequacy was defined as providing all levels of nutrients considered adequate for most young children by the EFSA [8], referred to below as reference values (RVs).

### 2.5. Statistical Analysis

#### 2.5.1. Comparison of Characteristics and Observed Diets in the 4 Groups of Children

Differences between the four groups of children were tested using the chi-squared test or logistic regression for categorical variables and general linear models for continuous variables.

#### 2.5.2. Diets Modeled with “Repertoire-Only Foods”

The percentage of children for whom it was possible to model a nutritionally-adequate diet from their repertoire-foods only was calculated and compared across the four groups using the chi-squared test or logistic regression. A sensitivity analysis was performed to assess whether or not the feasibility of modeling adequate diets was impacted by removing the nutrient constraints corresponding to the least frequently attained RVs.

#### 2.5.3. Diets Modeled with “All-Foods”

For “all-foods models”, variations of food weights between observed and modeled diets (mean weights of foods to increase, mean weights of foods to decrease and mean weights of non-repertoire-foods added, in grams) were also compared between groups of children. To examine whether or not variations in food quantities were significantly different from 0, paired Student *t*-tests were run for the whole sample and within each group of children. To determine whether these variations were similar across groups, a general linear model analysis was performed. A specific analysis focused on the variation in YCF and cow’s milk quantities between modeled and observed diets. Two-by-two comparison tests with Bonferroni corrections assessed, when relevant, statistically-significant differences between two groups.

The SAS system Version 9.4 (SAS Institute, Cary, NC, USA) was used for all analyses. When specified, analyses were adjusted for age and energy intake. A *p*-value < 0.01 was set as significant for the statistical tests.

## 3. Results

### 3.1. Description of the Sample

Children’s characteristics for the whole sample and for each of the four groups are presented in Table 1. The largest group was “no YCF, no Suppl” (61.6%), followed by “YCF, no Suppl” (29.7%), “no YCF, Suppl” (4.9%) and “YCF & Suppl” (3.7%). Gender, height, weight and BMI did not differ significantly across the four groups. Only 7.4% of children were still being breastfed at the time of the survey. Children in the “no YCF, no Suppl” group were slightly older (14.6 months) and had a higher energy intake (983 kcal/day) than those in the “YCF, no Suppl” group (13.8 months and 938 kcal/day). Diet quality indicators significantly differed between groups, with the lowest mean adequacy ratio and the highest PRSL observed in the “no YCF, no Suppl” group. The mean adequacy ratio was higher for children consuming supplements (92.5%), YCFs (94.8%) or both (97.5%) than for children in the “no YCF, no Suppl” group (88.6%).

### 3.2. Nutrients in Observed Diets

The RVs and the percentage of observed diets attaining each nutrient RV for the whole sample and across the four groups of children are presented in Table 2. Observed diets rarely reached the RV level for vitamin D (7.9%), fiber (20.6%), iron (28.2%), water (28.2%) and vitamin E (29.2%). For these nutrients and for most other nutrients, the percentage ranged significantly across groups. It was generally higher for children consuming YCFs and/or supplements. Almost all children’s diets (99.9%) supplied protein in sufficient amounts. Fewer than half of the children had fat intakes within the recommended range. The fat intakes of those not meeting the recommendation were mainly below the minimal recommended level. This was critical in the “YCF & Suppl” group, in which 74% (i.e., 100–26) of the children consumed less than 35% of their energy from fats.

### 3.3. Feasibility of Repertoire-Only Models

**Figure 1:**
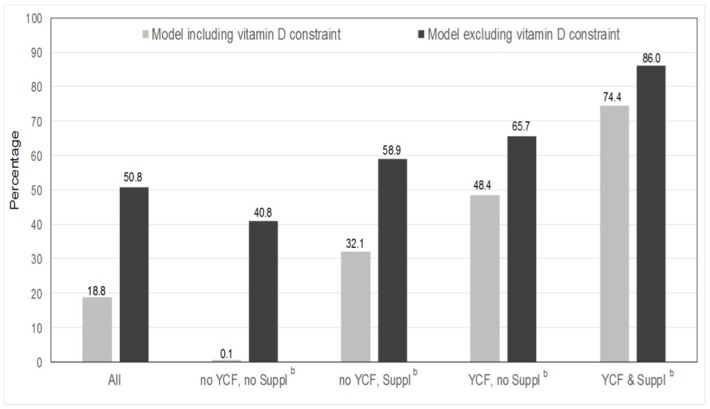
Percentage of children for whom it was feasible to model a nutritionally-adequate diet with their repertoire-foods only for the whole sample and across the four groups of children with and without vitamin D constraint ^a^^,b^. ^a^ Significant (*p* < 0.01) associations were observed between the percentage of feasibility and groups of children whatever the model (i.e., with and without the vitamin D constraint), with and without adjustments for age and energy (using logistic regression and chi-squared tests, respectively); ^b^ “no YCF, no Suppl” refers to children who did not consume either YCFs or supplements during the four days of dietary record; “no YCF, Suppl” refers to those who did not consume YCFs, but who consumed supplements; “YCF, no Suppl” refers to those who consumed YCFs, but not supplements; “YCF & Suppl” refers to those who consumed both YCFs and supplements.

When only repertoire-foods were allowed, achieving EFSA nutritional adequacy was almost impossible for children in the “no YCF, no Suppl” group (Figure 1). The percentage of feasibility in this group was 0.1% (only one child out of 707). By contrast, feasibility reached 74.4% in the “YCF & Suppl” group. As vitamin D was the least frequently attained RVs in the observed diets, it was removed from the sensitivity analysis. The percentage of feasibility consequently increased for each group of children.

### 3.4. Dietary Changes Induced by the All-Foods Models

**Figure 2:**
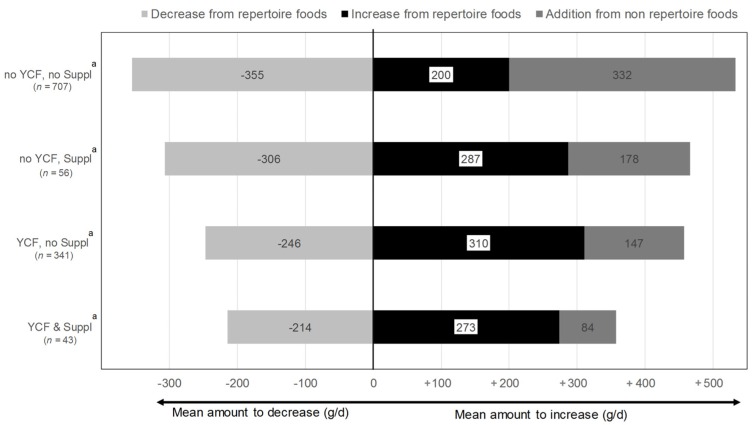
Variations in food quantities between diets modeled (all-foods models ^b^) and observed diets, across the four groups of children *^,^**. ^a^ “No YCF, no Suppl” refers to children who did not consume either YCFs or supplements during the four days of dietary record; “no YCF, Suppl” refers to those who did not consume YCFs, but who consumed supplements; “YCF, no Suppl” refers to those who consumed YCFs, but not supplements; “YCF & Suppl” refers to those who consumed both YCFs and supplements. ^b^ “All-foods models” refers to the linear programming models where all of the foods reported as consumed by the sample were taken as variables. * For each group of children, the mean difference between the total amount to increase and the total amount to decrease differed significantly from zero and was positive. ** The mean amount to decrease from repertoire-foods, the mean amount to increase from repertoire-foods and the mean amount to increase from non-repertoire-foods were significantly different across the four groups of children, with or without adjustment for age and energy.

When access to all foods (i.e., both repertoire- and non-repertoire-foods) was allowed, it was possible to model a nutritionally-adequate diet for each child (i.e., feasibility reached 100%). On average, this induced a net increase in total diet weight (on average +185 g/day), as a result of both the increase and decrease in repertoire-foods and the addition of non-repertoire-foods. The largest variations in food quantities were required for children of the “no YCF, no Suppl” group (Figure 2). By contrast, in the “YCF & Suppl” group, the addition of non-repertoire-foods and the decrease in repertoire-foods were significantly smaller than in all of the other groups (two-by-two tests, data not shown).

Quantities of food categories and subcategories for observed and modeled diets (all-foods models) are presented in Table 3. There were significant changes across groups between the observed and modeled diet for supplements, YCFs, cow’s milk, meat, eggs and animal fats. Supplements and YCFs increased significantly for all groups, except for “YCF & Suppl”, with the largest variation for the “no YCF, no Suppl”. In this group, almost all of the non-repertoire-food added (i.e., 332 g/day; Figure 2) was composed of YCF (+312 g/day). Cow’s milk significantly decreased in all of the groups except for “YCF & Suppl”, with the largest decrease in cow’s milk (−266 g/day) for the “no YCF, no Suppl” group. In the whole sample, besides the increase in YCF and the decrease in cow’s milk, the other important dietary shifts were increases in water (+110 g/day) and fruit and vegetables (+65 g/day). Starchy foods were decreased for all of the groups of children, except in “no YCF, Suppl”, while added fats were increased in all of the groups.

### 3.5. Variation of YCF and Cow’s Milk between Observed Diets and Those Modeled with All-Foods Models

**Figure 3:**
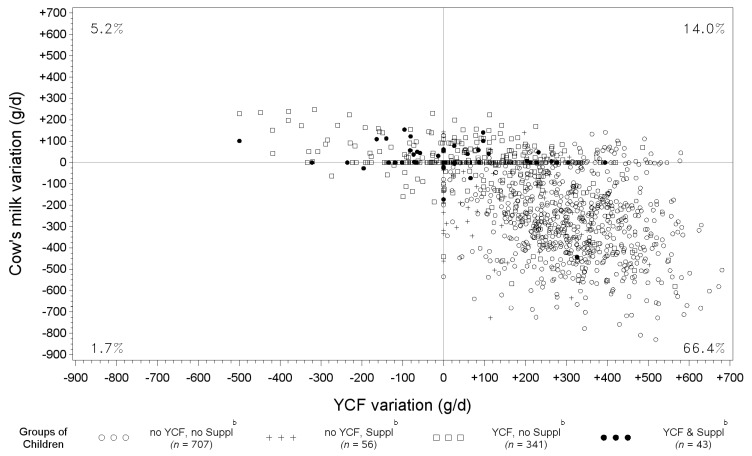
Scatter plot of the variations in YCF and cow’s milk quantities between diets modeled with all-foods models ^a^ and observed diets and percentage of children in each quarter *, according to the groups of children. ^a^ “All-foods models” refers to the linear programming models where all of the foods reported as consumed by the sample were taken as variables. ^b^ “No YCF, no Suppl” refers to children who did not consume either YCFs or supplements during the four days of dietary record; “no YCF, Suppl” refers to those who did not consume YCFs, but who consumed supplements; “YCF, no Suppl” refers to those who consumed YCFs, but not supplements; “YCF & Suppl” refers to those who consumed both YCFs and supplements. * Values in each quarter indicate the percentage of children in this quarter, excluding those with null variations. Null variations for both cow’s milk and YCFs (spots at the center of the grid) were seen for only 0.4% of the whole sample; 2.5% had a variation in cow’s milk, but not in YCF (spots on the vertical line, *x* = 0); and 9.6 % had a variation in YCF, but not in cow’s milk (spots on the horizontal line, *y* = 0).

Figure 3 shows, for each individual (represented by a spot), the variations of YCF and cow’s milk quantities between modeled and observed diets (g/day). For a large majority of children (66.4%), diet modeling induced a decrease in cow’s milk and an increase in YCF quantities. 

A statistically-significant negative correlation was found between cow’s milk and YCF variations (Pearson correlation coefficient = −0.62, *p* < 0.0001, after adjustment for age and energy intake). 

**Figure 4:**
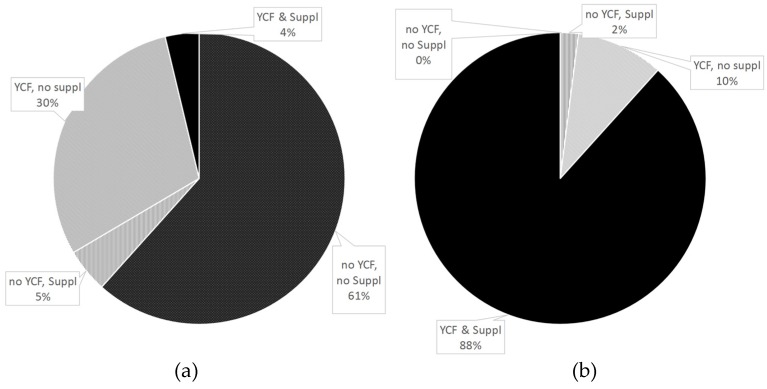
Percentage of diets containing YCF and/or supplement in (**a**) observed diets and (**b**) diets modeled with all-foods models ^a^. ^a^ “All-foods models” refers to the linear programming models where all of the foods reported as consumed by the sample were taken as variables.

Figure 4 shows the percentages of diets containing YCFs and/or supplements before (a) and after (b) optimization. Although most observed diets (61%) contained neither YCFs nor supplements, all modeled diets included either or both, there being no longer any “no YCF, no Suppl” diets after optimization.

## 4. Discussion

Based on an innovative approach taking individual dietary patterns into account, we confirmed our hypothesis that the consumption of YCFs and/or supplements is not strictly necessary to ensure nutritional adequacy. However, it proved almost impossible to ensure nutritional adequacy without introducing either YCFs or supplements.

Our study confirms the EFSA NDA Panel’s opinion that it is feasible to satisfy nutrient needs without consuming YCFs [8]. However, our results show that children who did not consume YCFs did not consume relevant food and drink alternatives able to provide critical nutrients. In addition, supplements, considered by the EFSA as an alternative means of providing critical nutrients, were rarely used. Inadequate dietary choices in the “no YCF, no Suppl” group were illustrated by lower diet quality indicators (a lower mean adequacy ratio and higher PRSL than in the other groups), low percentages of feasible adequate diets with repertoire-foods alone (one out of 707) and higher dietary changes than for children consuming YCFs, supplements or both in all-foods models. In this “no YCF, no Suppl” group, the main dietary shifts needed to meet nutritional adequacy consisted of introducing an average of 312 g of YCF, comparable to the intake level of 300 mL suggested by the British Nutrition Foundation [27], and reducing cow’s milk correspondingly by 266 g. However, we note that 25% of the children from the “YCF, Suppl” group still did not achieve nutritional adequacy in repertoire-only models, suggesting that YCFs or supplement are efficient, but not sufficient. Increasing the consumption of water (+110 g/day) and fruits and vegetables (+66 g/day) was necessary, whatever the consumption of YCF or supplement. These changes contributed to an increase in the average total diet weight in all of the groups of children and, therefore, to a decrease in the energy density of their diets.

The present study has limitations. The validity of the results obtained with diet modeling is dependent on the quality of input data and on the decisions made when building the models. The DNSIYC study indicates dietary intake over a short period. Infrequently-consumed foods may be under- or over-estimated, and this study cannot fully reflect children’s exact dietary habits. Some assumptions may be questioned. Imposing EFSA RVs on every child may not be justified. For most nutrients, RVs were population reference intake values or adequate intake values, although it is recommended that the average requirement be used to estimate the percentage of the population at risk of inadequate intake from a population’s usual intake distribution. Aiming to attain the RV will ensure that most individuals meet their nutrient requirements [28]. Nearly all modeled diets included YCFs and supplements, which may not be realistic, as compliance with supplement prescription is low even when these are freely provided to low income households [29], and the high cost of YCFs (compared with cow’s milk) may curtail their consumption. Further work could include diet cost as a constraint in the models. 

Individual diet modeling has been developed and applied to individual adult diets to explore the feasibility of meeting a set of nutrient recommendations and to quantify the minimum dietary shifts needed to reach nutritional adequacy [12,25,30,31]. As this is the first time that this powerful approach has been applied to test the dietary changes needed to meet nutritional recommendations for young children, it is crucial that other studies now address this question in different socio-cultural and geographical contexts and test different sets of nutrient recommendations.

The only study that has previously assessed the possibility of achieving nutritional adequacy without YCF is a German study highlighted by the EFSA NDA Panel on the role of YCFs [8]. In this earlier study, an optimized mixed diet without YCF and achieving reference intakes for 22 nutrients for children aged 1–18 years was developed [32]. The panel considered that this diet “can be taken as one example of dietary patterns which can ensure a sufficient energy and nutrient supply in infants and young children (except for vitamin D)” [8]. Our study qualifies these results, as it shows that achieving this adequacy is possible, but difficult. Compared with the previous study, it has some additional strengths: unlike Kersting et al., who based their conclusions on a single optimized diet, the present study is based on a total of 1147 generated optimized diets, which enhances the robustness of our results, as individual food habits were taken into account. The comparability of the German study with our results is hindered by differences in the target population (extrapolation of findings from 4–6 years old and 13–14 years old to 1–18 years old in the German study) and in nutrient requirement references (German reference nutrient intakes [33] and additional pediatric preventive recommendations [34] in the German study).

Our results are in line with several observational studies carried out among 12–24-month-old European children supporting the relevance of the nutrient content of YCFs in comparison with cow’s milk. For example, in France, Ghisolfi et al. [35] have shown that consuming 250 mL per day of cow’s milk (or more) increased the risks of insufficient intakes in alpha-linolenic acid, iron, vitamin C and vitamin D, whereas consuming the same amount of YCF reduced the risk of insufficiencies for the stated nutrients. In Ireland, Walton and Flynn indicated, by comparing two groups of Irish children (consumers of YCFs vs. consumers of unfortified cow’s milk), that YCFs accounted for nearly 80% and 45% of vitamin D and iron RVs, respectively, whereas cow’s milk contributed to less than 10% and 4%, respectively [36]. In Greece, 95% of children with iron deficiency anemia were found to drink cow’s milk, whereas 91% of children without iron deficiency anemia consumed fortified milks [37]. A recent simulation study showed that replacing habitual cow’s milk intake by YCF leads to nutritional intakes more in line with the recommendations in the U.K. in 12–18-month-old children [38].

Finally, following the set of nutritional constraints also induced improvements in PRSL. In particular, protein and sodium intakes were slightly decreased after diet modeling, mainly due to the decrease in cow’s milk, inducing a significant decrease in PRSL between observed and modeled diets, in both “no YCF” groups of children (in the “no YCF, no Suppl” group, PRSL decreased from 332 mmol to 302 mmol and in the “no YCF, Suppl” group, PRSL decreased from 328 mmol to 313 mmol, *p* < 0.001 in both groups). This is of particular importance because excessive sodium and protein levels in toddlers have been respectively associated with elevated blood pressure, a risk factor for cardiovascular and renal disease [39,40,41,42] and higher prevalence of being overweight and obesity in adults [43,44,45,46,47].

## 5. Conclusions

The present study helps to clarify the role of YCFs and/or supplements in ensuring that the nutrient needs of young children are covered. Increasing YCF and supplement consumption was found to be the shortest way to achieving European Food Safety Agency nutrient requirements for 12–18-month-old U.K. children. Besides increased YCF consumption, a simultaneous decrease in cow’s milk consumption was needed for most children to reach nutritional adequacy. This is the first study to assess individual dietary shifts needed to achieve nutritional adequacy in young children; other studies using a similar approach are now needed to explore this question further in other populations living in different socio-cultural and geographical contexts.

## Figures and Tables

**Table 1:** Gender, age, height, weight, BMI and diet characteristics (reported breastfeeding, energy intake and nutritional quality indicators) in the four groups of children *.

	All	No YCF, No Suppl ^a^	No YCF, Suppl ^a^	YCF, No Suppl ^a^	YCF & Suppl ^a^	*p* *
**Number of children (*n*)**	1147	707	56	341	43	
**Gender (%)**						
Male	50.9	51.6	53.6	49.6	46.5	0.83
Female	49.1	48.4	46.4	50.4	53.5	
**Child's age (in months)**						<0.001 ^1^
Mean	14.3	14.6	14.5	13.8	14.4	
SD	1.62	1.58	1.56	1.61	1.60	
**Anthropometric data**						
Infant height (cm)						
Mean	79.7	79.8	79.8	79.5	79.2	0.31
SD	3.46	3.50	3.35	3.24	4.52	
*n* missing	145	89	6	45	5	
Infant weight (kg)						
Mean	10.9	11.0	10.7	10.9	10.6	0.12
SD	1.31	1.33	1.22	1.29	1.31	
Number of missing values	42	27	2	10	3	
BMI (kg/m^2^)						
Mean	17.1	17.2	16.7	17.1	16.8	0.16
SD	1.56	1.54	1.54	1.62	1.52	
Number of missing values	158	96	7	49	6	
**Reported breastfeeding (%)**						
Still breastfeeding	7.41	8.49	14.3	4.40	4.65	0.02
No longer breastfeeding	68.3	64.6	67.9	74.8	79.1	0.004 ^1^
Never breastfed	24.1	26.7	17.9	20.8	16.3	0.06
Number of missing values	1	1	0	0	0	
**Energy (kcal/day)**						
Mean	968	983	972	938	950	0.005 ^1^
SD	196	199	228	183	166	
**Diet quality indicators**						
Mean adequacy ratio (%)						
Mean	90.9	88.6	92.5	94.8	97.5	<0.001 ^1,2,3,4,5^
SD	6.05	5.34	4.73	5.28	2.16	
PRSL (mmol)						
Mean	312	332	328	272	277	<0.001 ^1,3,5,6^
SD	78.8	76.2	87.3	66.6	66.9	

^a^ “no YCF, no Suppl” refers to children who did not consume either young child formulae (YCFs) or supplements during the four days of dietary record; “no YCF, Suppl” refers to those who did not consume YCFs, but who consumed supplements; “YCF, no Suppl” refers to those who consumed YCFs, but not supplements; “YCF & Suppl” refers to those who consumed both YCFs and supplements. * Statistical significance of the differences across the four groups of children. General linear model tests were run for all tests except “reported breastfeeding”, which was tested using the chi-squared test. ^1^ Indicates a significant difference between “no YCF, no Suppl” and “YCF, no Suppl”; ^2^ indicates a significant difference between “no YCF, no Suppl” and “no YCF, Suppl”; ^3^ indicates a significant difference between “no YCF, no Suppl” and “YCF & Suppl”; ^4^ indicates a significant difference between “YCF & Suppl” and “YCF, no Suppl”; ^5^ indicates a significant difference between “YCF & Suppl” and “No YCF, Suppl”; ^6^ indicates a significant difference between “YCF, no Suppl” and “no YCF, Suppl”.

**Table 2:** Reference values for each nutrient, percentage of observed diets attaining each reference value (RV) for the whole sample and across the four groups of children ^a^.

Nutrients	Reference Value	All (*n* = 1147)	No YCF, No Suppl ^a^	No YCF, Suppl ^a^	YCF, No Suppl ^a^	YCF & Suppl ^a^	*p*-Unadjusted Analysis *	*p*-Adjusted Analysis **
			(*n* = 707)	(*n* = 56)	(*n* = 341)	(*n* = 43)		
*Macronutrients*								
Water (H_2_O)	≥1100 mL/day ^b^	28.2	29.3	26.8	25.2	37.2	0.30	0.18
Proteins	≥1.14 g/kg body weight ^c^	99.9	100	100	99.7	100	0.50 ***	0.99 ***
Carbohydrates	45%–60% energy ^d^	82.0	80.9	83.9	85.6	69.8	0.04	0.03
Carbohydrates min	≥45% of energy ^d^	90.0	86.3	87.5	97.1	97.7	<0.001	<0.0001
Carbohydrates max	≤60% of energy ^d^	92.0	94.6	96.4	88.6	72.1	<0.001	<0.0001
Fiber	>10 g/day ^b^	20.6	15.3	10.7	30.8	39.5	<0.001	<0.001
Total fats	35%–40% energy ^d^	43.7	45.0	51.8	41.9	25.6	0.04	0.08
Total fats min	≥35% of energy ^d^	59.0	62.9	66.1	54.0	25.6	<0.001	<0.001
Total fats max	≤40% of energy ^d^	84.7	82.0	85.7	88.0	100	0.002	0.009
*n*-6 FA	≥3% energy ^f^	86.7	81.2	80.4	97.6	100.0	<0.001	<0.001 ***
*n*-3 FA	≥0.5% energy ^f^	77.0	68.3	69.6	93.5	97.7	<0.001	<0.001
*Vitamins*								
Thiamin	≥0.5 mg/day ^c^	95.1	94.1	98.2	96.2	100.0	0.12 ***	0.03 ***
Riboflavin	≥0.8 mg/day ^b^	93.1	93.5	96.4	90.9	100.0	0.08 ***	0.37 ***
Niacin	≥9 mg nicotinic acid eq/day ^c^	97.3	98.6	98.2	94.4	97.7	0.001 ***	0.007
Pantothenic acid	≥4 mg/day ^b^	77.3	83.0	89.3	63.3	79.1	<0.001	<0.001
Vitamin B6 min	≥0.7 mg/day ^c^	86.9	93.5	96.4	71.5	88.4	<0.001	<0.001
Vitamin B6 max	≤5 mg/day ^e^	100.0	100.0	100.0	100.0	100.0	N.A.	N.A.
Biotin	≥20 μg/day ^b^	60.1	66.0	67.9	48.4	44.2	<0.001	<0.001
Folates	≥100 μg/day ^c^	88.7	87.1	92.9	90.0	100.0	0.03	0.02 ***
Vitamin B12	≥0.9 μg/day ^c^	99.6	99.6	100.0	99.7	100.0	0.92 ***	0.91 ***
Vitamin C	≥20 mg/day ^c^	95.9	93.3	100.0	100.0	100.0	<0.001 ***	0.99 ***
Vitamin D min	≥10 μg/day ^b^	7.9	0	16.1	15.5	67.4	<0.001 ***	<0.001 ***
Vitamin D max	≤50 μg/day ^e^	100.0	100.0	100.0	100.0	100.0	N.A.	N.A.
Vitamin E	≥6 mg tocopherol eq/d ^b^	29.2	5.7	33.9	70.4	83.7	<0.001	<0.001
Retinol equivalent	≥400 μg /day ^b^	79.2	70.9	94.6	92.1	95.3	<0.001	<0.001
Retinol	≤800 μg/day ^e^	98.5	99.6	89.3	99.4	86.0	<0.001 ***	<0.001 ***
*Minerals*								
Sodium	≥170 mg/day ^b^	99.8	100.0	100.0	99.4	100.0	0.19 ***	0.97 ***
Potassium	≥800 mg/day ^c^	97.4	98.2	98.2	95.3	100.0	0.03 ***	0.05 ***
Magnesium	≥85 mg/day ^c^	93.3	95.3	94.6	89.1	90.7	0.002 ***	0.001 ***
Chloride	≥270 mg/day ^b^	100.0	100.0	100.0	100.0	100.0	N.A.	N.A.
Calcium	≥600 mg/day ^c^	78.2	82.6	76.8	69.2	79.1	<0.001	<0.001
Phosphorus	≥460 mg/day ^c^	94.2	96.0	96.4	90.0	93.0	0.001 ***	0.002 ***
Iodine min	≥90 µg/day ^b,c^	88.1	91.65	91.1	81.2	79.1	<0.001	<0.001
Iodine max	≤200 µg/day ^e^	62.1	49.1	50.0	87.7	88.4	<0.001	<0.001
Iron	≥8 mg/day ^b,c^	28.2	9.9	19.6	61.0	79.1	<0.001	<0.001
Copper min	≥0.4 mg/day ^c^	71.9	64.9	69.6	84.2	93.0	<0.001	<0.001
Copper max	≤1 mg/day ^e^	99.5	99.9	98.2	98.8	100.0	0.08 ***	0.13 ***
Zinc min	≥4 mg/day ^c^	91.6	89.1	89.3	96.2	100.0	<0.001 ***	<0.001 ***
Zinc max	≤7 mg/day ^e^	91.2	97.2	91.1	81.8	67.4	<0.001 ***	<0.001 ***
Selenium min	≥20 µg/day ^c^	59.4	55.3	60.7	66.3	69.8	0.004	<0.001
Selenium max	≤60 µg/day ^e^	100.0	100.0	100.0	100.0	100.0	N.A.	N.A.
Manganese	≥0.5 mg/day ^b^	96.4	97.0	100.0	94.4	97. 7	0.07 ***	0.94 ***

^a^ “no YCF, no Suppl” refers to children who did not consume either YCFs or supplements during the four days of dietary record; “no YCF, Suppl” refers to those who did not consume YCFs, but who consumed supplements; “YCF, no Suppl” refers to those who consumed YCFs, but not supplements; “YCF & Suppl” refers to those who consumed both YCFs and supplements. ^b^ Requirement derived by the EFSA from an adequate intake. ^c^ Requirement derived by the EFSA from a Population Reference Intake. ^d^ Requirement derived by the EFSA from a reference intake range. ^e^ Requirement derived by the EFSA from a tolerable upper intake level. ^f^ Based on Nordic recommendations [26]. * Unadjusted analysis, using a chi-squared test for the percentage of observed diets attaining each RV; ** analysis adjusted for age and energy intake, using logistic regression for the percentage of observed diets attaining each RV; *** *p*-value to be interpreted with caution due to the presence of 100% or 0% in one group.

**Table 3:** Food categories and subcategories quantities (g/day) in observed diets and diets modeled with the all-foods models ^a^, for the four groups of children.

	All ^1^ (*n* = 1147)				No YCF, No Suppl ^b^^2^ (*n* = 707)				No YCF, Suppl ^b^^3^ (*n* = 56)				YCF, No Suppl ^b^^4^ (*n* = 341)				YCF & Suppl ^b^^5^ (*n* = 43)				Test of Modeled vs. Observed Variation Across Groups	
	Observed		Modeled		Observed		Modeled		Observed		Modeled		Observed		Modeled		Observed		Modeled		Unadjusted *p*	Adjusted *p* **
	Mean	SD	Mean	SD	Mean	SD	Mean	SD	Mean	SD	Mean	SD	Mean	SD	Mean	SD	Mean	SD	Mean	SD		
**Supplements ^1,2,3,4^**	0.11	0.54	0.74	1.03	0.00	0.00	0.81	1.03	1.30	1.34	1.82	1.55	0.00	0.00	0.36	0.59	1.29	1.37	1.35	1.36	<0.001	**<0.001**
**Dairy products ^1,2,3,4^**	503	185	529	134	484	185	512	128	519	207	441	157	536	178	577	126	539	177	551	124	0.001	**<0.001**
YCF ^1,2,3,4^	119	200	345	154	0.00	0.00	312	132	0.00	0.00	157	135	353	186	439	143	383	190	399	155	<0.001	**<0.001**
Cow’s milk ^1,2,3,4^	303	228	122	87.9	399	200	133	86.9	413	203	192	125	113	142	88.2	69.3	88.8	117	106	76.9	<0.001	**<0.001**
Breast milk	20.7	89.0	20.7	89.0	24.1	94.5	24.1	94.5	46.2	155	46.2	155	11.6	63.1	11.6	63.1	4.65	21.3	4.65	21.3		
Fresh dairy products ^1,2,3,4,5^	51.5	40.0	33.9	33.1	52.0	40.3	34.7	33.6	51.6	39.1	38.2	34.0	50.5	39.3	31.8	32.1	51.7	43.1	32.7	31.8	0.73	0.79
Cheese and cream ^1,2,4^	8.49	9.71	7.49	8.66	8.47	9.69	7.75	8.81	8.22	9.87	7.19	8.46	8.23	9.44	6.76	7.97	11.1	11.6	9.43	11.1	0.22	0.32
**Fruit and vegetable ^1,2,3,4,5^**	156	99.0	222	91.1	161	97.2	223	90.3	185	115	268	94.2	139	96.0	211	88.9	167	112	228	98.4	0.03	0.10
Fruits ^1,2,3,4^	78.5	63.0	94.7	65.6	81.3	62.3	94.6	64.5	95.2	73.7	116	67.1	69.5	60.2	91.8	66.5	81.1	73.1	92. 6	69.8	0.02	0.02
Vegetables ^1,2,3,4,5^	56.1	44.9	106	52.5	56.7	45.1	105	51.9	69.3	51.3	132	53.3	51.9	43.0	103	53.4	62.3	44.5	115.3	46.6	0.09	0.04
Fruit juice	13.0	38.7	12.4	33.1	13.2	37.7	13.2	33.8	13.3	33.3	12.4	30.5	11.4	39.6	10.1	31.1	22.1	52.4	17.8	39.0	0.77	0.90
Soups	8.63	25.3	8.37	23.9	10.3	28.8	10.2	27.9	7.45	12.6	7.29	12.4	6.20	19.6	5.48	16.3	1.36	5.63	2.36	9.16	0.48	0.39
**Starchy foods ^1,2,4,5^**	119	60.8	105	60.0	128	60.4	113	60.7	118	56.4	115	63.7	101	57.9	88. 9	54.3	111	66.7	94.9	60.5	0.13	0.08
Bread ^1,2,4,5^	27.9	19.8	20.6	17.4	30.9	21.0	23.2	18.6	21.4	13.9	19.1	14.1	24.0	17.6	16.6	14.5	18.2	13.0	13.1	12.5	0.03	0.02
Other starchy foods ^1,2^	91.0	57.1	84.7	54.8	97.0	56.2	89.9	55.0	96.6	55.5	95.8	59.1	77.5	55.9	72.3	51.46	92.7	67.2	81.7	58.4	0.40	0.34
**Meat fish eggs ^1,2,3,4,5^**	51.5	34.8	60.5	34.7	54.9	35.3	64.9	34.9	49.2	30.4	64.5	32.6	45.5	32.1	51.4	31.6	47.3	44.9	55.5	44.2	<0.001	**0.001**
Meat ^3^	34.6	28.6	35.1	27.9	38.1	30.1	38.3	29.0	27.5	21.1	33.4	25.3	28.9	24.6	29.4	24.2	30.1	31.9	30.7	31.8	<0.001	**<0.001**
Fish ^1,2^	9.77	14.1	10.9	14.5	9.91	14.3	11.2	14.5	11.3	16.1	11.3	17.4	9.33	13.4	10.2	14.1	9.07	13.4	10.4	14.6	0.65	0.77
Eggs ^1,2,3,4,5^	7.20	11.5	14.5	17.8	6.87	11.3	15.4	19.0	10.5	14.3	19.7	19.2	7.23	11.7	11.8	14.8	8.18	9.42	14.4	15.3	<0.001	**<0.001**
**Sweets & salted foods and drinks ^1,2,4^**	74.5	128	65.0	120	88.6	146	77.6	138	70.1	112	67.8	110	48.5	79.4	41.1	76.3	52.8	83.5	44.9	72.2	0.06	0.05
Savory foods ^1^	2.16	3.78	2.00	3.62	2.51	3.99	2.35	3.84	1.76	3.91	1.81	3.99	1.57	3.21	1.38	2.93	1.60	3.65	1.51	3.64	0.86	0.75
Soft drinks ^1,2^	42.0	120	39.3	115	52.9	140	48.9	133	42.2	107	41.7	107	21.4	70.0	20.9	69.4	25.8	64.9	24.0	64.1	0.09	0.16
Sweet foods ^1,2,4^	30.3	31.4	23.7	25.7	33.2	30.6	26.3	26.7	26.1	26.1	24.2	25.4	25.5	30.3	18.8	22.8	25.5	48.7	19.4	25.7	0.29	0.24
**Toddlers foods and drink ^1,2,4^**	62.9	108	57.0	91.9	41.9	83.1	37.9	69.4	78.4	118	74.8	103	94.4	129	85.5	110	138	170	123	144	0.18	0.36
Toddlers foods	52.7	85.1	50.9	81.9	35.6	65.8	34.5	63.0	67.7	90.6	68.2	92.9	81.4	107	77.8	103	86.2	92.5	83.5	89.1	0.49	0.78
Toddlers drinks ^1,2^	10.2	51.4	6.15	34.6	6.32	41.6	3.31	27.9	10.8	47.4	6.55	25.2	13.0	56.1	7.72	36.0	52.3	111	39.9	83.8	0.28	0.34
**Added fats ^1,2,3,4,5^**	4.80	4.17	6.34	5.01	5.14	4.31	6.75	5.10	4.55	3.39	7.04	4.70	4.34	4.04	5.39	4.71	3.30	2.83	6.14	5.12	0.01	0.03
Animal fats ^1,5^	1.70	3.37	1.92	3.62	1.67	3.43	1.85	3.60	1.64	2.47	1.86	2.88	1.83	3.49	1.94	3.62	1.35	2.25	2.92	4.72	0.005	**0.003**
Vegetable fats ^1,2,3,4,5^	3.10	3.46	4.42	4.38	3.47	3.65	4.90	4.53	2.91	3.13	5.18	4.57	2.51	3.12	3.46	3.94	1.96	2.22	3.23	3.28	0.03	0.16
**Water ^1,2,3,4,5^**	121	129	231	154	119	134	223	162	158	125	301	152	116	120	235	137	141	124	238	118	0.04	0.09
**Low calorie drinks, tea and coffee**	97.8	174	97.3	173	122	197	121	196	32.7	70.9	32.7	70.9	67.4	130	67.4	130	23.2	51.7	23.2	51.7	0.67	0.73
Low calorie drinks	93.9	172	93.4	171	118	195	117	193	29.3	68.3	29.3	68.3	64.0	128	64.0	128	19.6	51.6	19.6	51.6	0.67	0.73
Tea coffee	3.89	20.4	3.89	20.4	4.20	22.1	4.20	22.1	3.44	13.4	3.44	13.4	3.37	18.4	3.37	18.4	3.58	12.3	3.58	12.3	0.89	0.89
**Others ^c,1,2,3,4^**	7.27	11.1	8.95	12.5	7.95	10.7	10.1	12.6	5.71	8.83	7.64	9.91	6.48	12.4	7.35	12.9	4.27	8.70	5.06	9.11	0.001	0.01

^a^ “All-foods models” refers to the linear programming models where all of the foods reported as consumed by the sample were taken as variables; ^b^ “no YCF, no Suppl” refers to children who did not consume either YCFs or supplements during the four days of dietary record; “no YCF, Suppl” refers to those who did not consume YCFs, but who consumed supplements; “YCF, no Suppl” refers to those who consumed YCFs, but not supplements; “YCF & Suppl” refers to those who consumed both YCFs and supplements; ^c^ others include savory sauces, pickles, gravies, condiments, powders for drinks and wine; ^1^ indicates a significant difference (*p* < 0.01) between food quantities from observed and modeled diets in the overall sample; ^2^ indicates significant difference (*p* < 0.01) between food quantities from observed and modeled diets in the “no YCF no Suppl” group of children; ^3^ indicates significant difference (*p* < 0.01) between food quantities from observed and modeled diets in the “no YCF Suppl” group of children; ^4^ indicates significant difference (*p* < 0.01) between food quantities from observed and modeled diets in the “YCF no Suppl” group of children; ^5^ indicates significant difference (*p* < 0.01) between food quantities from observed and modeled diets in the “YCF & Suppl” group of children; ** analysis adjusted for age and energy intake, using the general linear model for variation between modeled and observed quantities across groups.

## Supplementary Materials

The following are available online at http://www.mdpi.com/2072-6643/8/9/539/s1, Table S1: Role of Young Child Formulae and Supplements to Ensure Nutritional Adequacy in UK Young Children.

## Appendix A. Individual Diet Modeling with Linear Programming

### Appendix A.1. Principle of Diet Modeling

Linear programming is a mathematical optimization tool. In this study, it was used to find the shortest way to reach nutritional adequacy by minimizing the diet changes needed in the observed diet to satisfy all nutritional recommendations. More specifically, the objective function was the deviation from the existing diet; food weights were used as decision variables and nutritional requirements as constraints. More technically, a linear programming model is defined by a list of (food) variables, a set of constraints and an objective function [48]. In diet modeling, the food variables are the food intakes (in grams) that can be changed to design a diet at the lowest (minimization) or the highest (maximization) value of the objective function, while meeting all of the imposed constraints. When the constraints cannot be met with the food variables allowed, the model is mathematically unfeasible, and no modeled diet can be designed. A previous study showed that it is not always possible to find a combination of foods satisfying a comprehensive set of nutritional and acceptability constraints with only the repertoire-foods of a given individual [25].

### Appendix A.2. Types of Models Defined by Two Sets of Food Variables

In the present study, two linear programming models were run for each child, each differing in the list of the food variables allowed. “Repertoire-only models” allowed the inclusion of only repertoire-foods. “All-foods models” allowed both repertoire-foods and non-repertoire-foods, i.e., all foods recorded in at least one food diary of the survey. “Repertoire-only models” were used to assess the feasibility of designing nutritionally-adequate diets with repertoire-foods only, while “all-foods models” were used to identify the food changes needed to achieve nutritional adequacy [12].

### Appendix A.3. Nutritional and Acceptability Constraints

Nutritional and acceptability constraints were the same for both types of models. The energy content of each modeled diet was set equal to that of the corresponding observed diet. Nutritional adequacy was defined as the attainment of a set of 30 nutrient RVs based on the EFSA NDA Panel report. This report defines the nutrient levels considered adequate for most infants and young children [8]. For *n*-6 and *n*-3 fatty acids, for which no RVs were provided in the EFSA NDA Panel report, Nordic nutrient recommendations [26] were used. Minimum content of heme iron and maximum contents of sodium and added sugars were set to the corresponding observed intakes. Added sugars were defined as total non-milk extrinsic sugars minus non-milk extrinsic sugars from fruit juices [49]. Quantities of breast milk were set equal to the observed quantities in the corresponding modeled diets, as it would be hard to realistically impact the amount of breast milk a mother could give her child. Gender-specific acceptability constraints were also introduced. This imposed that the amount of foods, food subcategories and food categories in the modeled diets stayed below the 97.5th percentiles of the observed diets (except for foods included in the “low calorie drinks, tea and coffee” category, for which maximum quantities were set to the observed quantities).

### Appendix A.4. Objective Function

As previously described [12,25], the objective function aimed to design modeled diets as close as possible, in terms of food content, to the observed diet of each child. More technically, for “repertoire-only models”, the objective function minimized the sum of the absolute negative deviations between the modeled and observed quantities of each repertoire-food. For “all-foods models”, the same objective function was used for foods already consumed by the children, and the sum of non-repertoire-food quantities was added as an additional term to the objective function [12]. Thus, foods already consumed by the child were favored, and non-repertoire-foods were introduced only when necessary. In this case, the most frequently-consumed foods were favored.
