# Supplementary material for: Role of Young Child Formulae and Supplements to Ensure Nutritional Adequacy in U.K. Young Children
Source: Nutrients. 2016 Sep 2;8(9):539. doi: 10.3390/nu8090539 (PMC5037526; doi:10.3390/nu8090539)
Supplement: Supplementary file 1 [file nutrients-08-00539-s001.docx]

Supplementary Materials: Role of Young Child Formulae and Supplements to Ensure Nutritional Adequacy in UK Young Children

Florent Vieux, Chloé M. C. Brouzes, Matthieu Maillot, André Briend, Régis Hankard, Anne Lluch and Nicole Darmon

**Table S1.** Food categories and subcategories (including number of foods) used in the present study, in comparison with DNSIYC categorization.

| **New Categories** | **New Subcategories  (Number of Foods)** | **DNSIYC Categorization** |
| --- | --- | --- |
| Supplements | Supplements (23) | Dietary supplements |
| Dairy products | YCF (17) | Infant formula ^a^ |
|  | Cow’s milk (26) | 1% fat milk |
|  |  | Other milk ^a^ |
|  |  | Semi-skimmed milk |
|  |  | Skimmed milk |
|  |  | Whole milk |
|  | Breast milk (1) | Breast milk |
|  | Fresh dairy products (50) | Yogurt fromage frais and dairy desserts |
|  | Cheese and cream (62) | Cream (including imitation cream) ^a^ |
|  |  | Cheese |
| Fruit and vegetable | Fruit (158) | Fruit |
|  |  | Nuts and seeds |
|  | Fruit juice (30) | Fruit juice |
|  |  | Smoothies 100% fruit and/or juice |
|  | Soups (49) | Miscellaneous |
|  | Vegetables (225) | Salad and other raw vegetables |
|  |  | Vegetables not raw |
| Starchy foods and dishes | Bread (76) | Brown granary and wheatgerm bread |
|  |  | Other bread |
|  |  | White bread |
|  |  | Wholemeal bread |
|  | Other starchy foods (270) | Chips fried & roast potatoes  and potato products |
|  |  | High fiber breakfast cereals |
|  |  | Other breakfast cereals |
|  |  | Other potatoes potato salads & dishes |
|  |  | Pasta rice and other cereals |
| Meat fish eggs dishes | Eggs and eggs dishes (42) | Eggs and egg dishes |
|  | Fish and Fish dishes (92) | Other white fish shellfish & fish dishes |
|  |  | White fish coated or fried |
|  |  | Oily fish |
|  | Meat and meat dishes (333) | Bacon and ham |
|  |  | Beef veal and dishes |
|  |  | Burgers and kebabs |
|  |  | Chicken and turkey dishes |
|  |  | Coated chicken |
|  |  | Lamb and dishes |
|  |  | Liver & dishes |
|  |  | Meat pies and pastries |
|  |  | Other meat and meat products |
|  |  | Pork and dishes |
|  |  | Sausages |
| Sweets and salted foods and drinks | Savoury foods (26) | Crisps and savory snacks |
|  | Soft drinks (44) | Soft drinks not low calorie |
|  | Sweet foods (353) | Biscuits |
|  |  | Buns cakes pastries & fruit pies |
|  |  | Chocolate confectionery |
|  |  | Ice cream |
|  |  | Puddings |
|  |  | Sugar confectionery |
|  |  | Sugars preserves and sweet spreads |
| Toddlers foods and drinks | Toddlers drinks (5) | Commercial toddlers drinks ^a^ |
|  | Toddlers foods (107) | Commercial toddlers foods ^a^ |
| Added fats | Animal fats (5) | Butter |
|  | Vegetable fats (40) | Low fat spread |
|  |  | Other margarine fats and oils |
|  |  | Pufa margarine & oils |
|  |  | Reduced fat spread |
| Other foods drinks | Other foods & drinks (140) | Artificial sweeteners |
|  |  | Beer lager cider & perry |
|  |  | Miscellaneous |
|  |  | Wine |
| Low calorie drinks tea and coffee | Low calorie drinks (27) | Soft drinks low calorie |
|  | Tea coffee (10) | Tea coffee ^a^ |
| Water | Water (4) | Bottled water still or carbonated ^a^ |
|  |  | Tap water only ^a^ |

^a^ Indicates a name of DNSIYC subgroup.
